# Synthesis of Cu_3_P/SnO_2_ composites for degradation of tetracycline hydrochloride in wastewater†

**DOI:** 10.1039/d1ra05905j

**Published:** 2021-11-12

**Authors:** Huancong Shi, Tao Zheng, Yuanhui Zuo, Qiming Wu, Yun Zhang, Yi Fan, Paitoon Tontiwachwuthikul

**Affiliations:** Department of Environmental Science and Engineering, University of Shanghai for Science and Technology Shanghai 200093 P. R. China hcshi@usst.edu.cn; Huzhou Institute of Zhejiang University Huzhou Zhejiang 313000 P. R. China 1810321@tongji.edu.cn; Clean Energy Technology Research Institute (CETRI), Faculty of Engineering and Applied Science, University of Regina 3737 Wascana Parkway Regina Saskatchewan S4S 0A2 Canada; College of Environmental Science and Engineering, Tongji University Shanghai 200092 China

## Abstract

Antibiotic drugs have become dominating organic pollutants in water resources, and efficient removal of antibiotic drugs is the priority task to protect the water environment. Cu_3_P/SnO_2_ photocatalysts of various Cu_3_P loadings (10–40 wt% Cu_3_P) were synthesized using a combination of hydrothermal synthesis and a partial annealing method. Their photocatalytic activity was tested for tetracycline hydrochloride (TC-HCl) degradation under visible light irradiation. Cu_3_P/SnO_2_ samples were characterized by X-ray diffraction (XRD), N_2_-adsorption, ultraviolet-visible diffuse reflectance spectra (UV-vis DRS), scanning electron microscopy (SEM) and electrochemical impedance spectroscopy (EIS). The results showed that the p–n type heterostructure between Cu_3_P and SnO_2_ was successfully constructed, and addition of Cu_3_P to SnO_2_ could improve its photocatalytic activity at an optimized loading of 30 wt% Cu_3_P. In photocatalytic degradation studies, removal rates of around 80% were found in 30 minutes of dark reaction and 140 min of photodegradation. The removal rate was superior to that of Cu_3_P and SnO_2_ alone under the same experimental conditions. According to trapping experiments and electron spin resonance (ESR) measurements, photogenerated holes (h^+^) and superoxide radicals ˙O_2_^−^ were considered as the main oxidation species in the present system. Finally, the reuse experiments showed high stability of Cu_3_P/SnO_2_. This study reports Cu_3_P as a cocatalyst combined with semiconductor SnO_2_ to form a highly efficient heterogeneous photocatalyst for the degradation of tetracycline hydrochloride for the first time.

## Abstract 2

Cu_3_P@SnO_2_ was synthesized with Cu_3_P nanoparticles loaded on the surface of hollow SnO_2_ microspheres *via* a simple annealing process.

## Graphical abstract

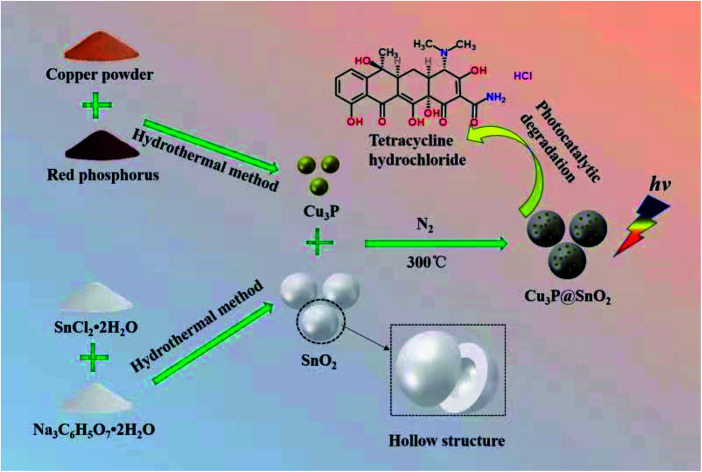

## 1. Introduction

Recently, antibiotics have been widely misused in medical treatment as antimicrobial drugs. Due to their coupling with the food chain, they bioaccumulate at lower and higher nutrient levels, resulting in a long-term negative impact on the environment.^1,2^ Tetracycline hydrochloride (TC-HCl) is a broad-spectrum antibiotic of tetracycline, which is widely used medically to prevent bacterial diseases of livestock, poultry and aquatic products.^3^ Its worldwide extensive misuse accelerates it accumulation in the environment.^4^ Because of its obvious mutagenic and teratogenic effects, it has drawn extensive attention.^5,6^

Therefore, it is particularly important to remove TC-HCl in wastewater. Compared with advanced oxidation methods, biological treatment methods, membrane filtration methods and adsorption methods,^7–12^ the photocatalytic degradation method is commonly used in the treatment of refractory organic wastewater due to its advantages of high efficiency, low energy consumption, and green environmental protection.^13^ The photocatalyst generates free radicals under light conditions and effectively decomposes organic chemicals in wastewater.^14–17^ Many semiconductor photocatalysts have been developed for decades. For example, Shen *et al.* prepared hierarchical carbon nitride photocatalyst,^18^ Song *et al.* constructed KCl/NH_4_Cl/g-C_3_N_4_ composite photocatalyst,^19^ Mao *et al.* prepared Bi_2_WO_6_/CuS composite photocatalyst,^20^ these catalysts have been used to degrade TC-HCl, and have achieved good degradation effects.

From the reports of photocatalytic materials in last decades, SnO_2_ as a photocatalyst has drawn extensive attention due to its excellent optical properties,^21^ electrical properties,^22^ and stable gas–sensitive properties.^23^ As an n-type semiconductor, SnO_2_ has a quite wide band gap of about 3.6 eV,^24^ which can only be excited by ultraviolet light with a wavelength of less than 400 nm, and its absorption efficiency is low under sunlight.^25^ The recombination rate of photogenerated electron–hole is high, which restricts its direct application in photocatalysis. Most researches of SnO_2_ as a photocatalytic material involved coupling SnO_2_ with other narrow-band semiconductors, or adding dopants to expand the light absorption to the visible light range.^26,27^ Therefore, there were multiple researches reported the photocatalytic properties of SnO_2_ doped metallic elements such as cobalt-doped SnO_2_ nanoparticles, Au/SnO_2_ composites and Ag/SnO_2_ composites^28–30^ or non-metallic elements such as carbon-doped SnO_2_ nanostructures and Cl-doped SnO_2_ photocatalysts.^31,32^ Some publications reported SnO_2_ coupled with other narrow-band semiconductors such as AgBr/SnO_2_ composites and Co_3_O_4_/SnO_2_ composites,^24,33,34^ and SnO_2_ coupled with wideband semiconductors such as TiO_2_ (ref. 35) or ZnO.^36,37^

Recently, studies have found that materials such as transition metal phosphides (MP) and their composites have excellent catalytic performance for hydrogen evolution reactions. For example, Gai *et al.* used Ni_2_P^38^ and Cao *et al.* used CoP^39^ to improve the hydrogen evolution efficiency of CdS. In addition, Sun *et al.* prepared a Cu_3_P/g-C_3_N_4_ heterostructure^40^ for photocatalytic hydrogen production. As a p-type semiconductor, Cu_3_P has a band gap of about 1.5 eV.^41^ If the p-type semiconductor is combined with the n-type semiconductor, an internal electric field is generated in the p–n junction area, which can efficiently facilitate the separation of photogenerated electrons and holes.^42^ Ioannidi *et al.* synthesized Cu_3_P/BiVO_4_ composite materials^43^ to degrade sulfamethoxazole in aqueous media as similar study. It is worth noting that, Cu_3_P-based composite materials have not been used as a photocatalyst to degrade TC-HCl yet based on literature study. This study filled the gap into the area.

The purpose of this work is to synthesize Cu_3_P/SnO_2_ heterostructure with different Cu_3_P loading in order to explore its catalysis and characteristics as a heterogeneous photocatalytic composite material in area of TC-HCl decomposition. We evaluated the photocatalytic activity of Cu_3_P/SnO_2_ sample for degradation of TC-HCl under visible light irradiation. Finally, the photocatalytic activity results of Cu_3_P/SnO_2_ composite material were compared with that of the self-made parent material of Cu_3_P or SnO_2_ under the same experimental conditions. The results may provide an alternative method for preparation of photocatalysts, under the case of wide band gap SnO_2_ semiconductors used.

## 2. Experiment section

### 2.1. Photocatalyst preparation

#### 2.1.1. Synthesis of SnO_2_ hollow microspheres

Hollow SnO_2_ microspheres were prepared by hydrothermal method.^44^ SnCl_2_·2H_2_O of 0.4890 g and a certain amount of Na_3_C_6_H_5_O_7_·2H_2_O were dissolved in distilled water of 12.5 mL at different molar ratios of 1 : *x* (*x* from 3 to 6) to generate homogeneous solution A. NaOH of 0.30 g was dissolved in 12.5 mL distilled water to generate homogeneous solution B. Solution B was slowly added to solution A, with the mixture continuously stirred for 24 hours. The transparent solution stirred was transferred to a 50 mL reaction kettle and placed in an oven at 180 °C for 12 hours. After cooling of the reaction kettle, the white precipitate at the bottom was alternately washed with ethanol and distilled water repeatedly. The collected samples were dried overnight in an oven at 60 °C to produce white SnO_2_ sample. The SnO_2_ samples prepared with different ratios of raw materials were labeled as SnO_2_-1 : 3, SnO_2_-1 : 4, SnO_2_-1 : 5 and SnO_2_-1 : 6 respectively.

#### 2.1.2. Synthesis of Cu_3_P

The 0.189 g (0.003 mol) copper powder and 0.3097 g (0.01 mol) red phosphorus were thoroughly ground evenly with an agate mortar. The mixed powder was collected in a beaker, and added with 80 mL deionized water. After the solution was continuously stirred for 0.5 h, the solution was transferred to a 100 mL reaction kettle and kept in an oven at 200 °C for 24 h. After cooling of the reaction kettle, the precipitate were centrifugally washed with carbon disulfide (CS_2_), ethanol and deionized water one by one, and the resulting products were dried overnight in the oven at 60 °C.

#### 2.1.3. Synthesis of Cu_3_P/SnO_2_ heterostructure

0.03 g Cu_3_P and 0.30 g SnO_2_ were sufficiently ground, and the mixture was transferred to 30 mL deionized water containing 0.25 g polyvinyl pyrrolidone (PVP). After stirring with ultrasonic for 24 hours, the precipitate was rinsed for many times with deionized water. Then it was dried, ground, and put into an atmosphere tube furnace at 300 °C first, and then annealed for 3 hours by introducing N_2_ to produce Cu_3_P/SnO_2_ sample with content of 10%. In addition, by varying the loading of Cu_3_P, the Cu_3_P/SnO_2_ heterostructure composite catalysts of 20%, 30% and 40% wt load were synthesized by the same preparation method, labeled as Cu_3_P/SnO_2_-1, Cu_3_P/SnO_2_-2, Cu_3_P/SnO_2_-3 and Cu_3_P/SnO_2_-4 respectively.

### 2.2. Characterization methods

The characterization of samples was carried out to understand their structure, surface, morphology, optical and photocatalytic properties. X-ray diffraction XRD was applied to analyze the phase composition of the prepared samples, with D/MAX-2500 diffractometer (Bruker test in Germany). The morphological characteristics were studied by scanning electron microscope (SEM), from Hitachi (S-4800). Ultraviolet-visible diffuse reflectance spectrometer of Japan Shimadzu Company's UV 2600 model was used to analyze the absorption properties of the prepared samples with BaSO_4_ as a reference in the wavelength range of 250 nm to 800 nm. The N_2_ adsorption and desorption isotherm test uses the Quantachrome company (NOVA 2200e) pore size analyzer. The specific surface area and pore size characteristics of the sample are calculated using Brunauer–Emmett–Teller (BET) and Barret–Joyner–Halenda (BJH) theoretical models, respectively.

### 2.3. Electrochemical test

The electrochemical impedance spectroscopy (EIS) test was performed on the electrochemical workstation of Shanghai Chenghua Instrument Co., Ltd. (CHI660E), China. Calomel electrode and platinum electrode were used for reference electrode and auxiliary electrode respectively. 0.5 M H_2_SO_4_ aqueous solution is used as the electrolyte, and copper electrode sample is used as the working electrode.

The electrode sample preparation process is as follows: 0.16 g polyvinylidene fluoride (PVDF) binder were mixed with 0.02 g catalyst sample and 0.02 g carbon black into a small glass jar. Then we add 500 μL *N*-methyl pyrrolidone to the mixture, and stir. After a few hours, the paste liquid is consistently coated on a copper sheet of 10 × 20 mm in size, and the other side of the copper sheet is pasted with insulating glue. The specific process of the electrochemical workstation was as follows: the three-electrode system and the electrochemical workstation were connected, then the open circuit potential of the electrode was tested, and later the initial voltage is set according to the open circuit potential. The SnO_2_ open circuit potential tested in this experiment is 0.006 V. The measured open circuit potentials of Cu_3_P/SnO_2_-1, 2, 3 and 4 samples are 0.010 V, 0.049 V, 0.055 V, and 0.058 V in sequence. The initial voltage E was set according to open circuit potential value of tested sample, with other parameters remain unchanged.

### 2.4. Photocatalytic activity experiments

The photocatalytic activity of the samples was systematically evaluated by visible light-driven degradation of TC-HCl. The photocatalytic experiments were performed under a 1000 W Xenon lamp at constant temperature 25 °C. The photocatalytic degradation activity of Cu_3_P/SnO_2_-1–4 were evaluated. First, 50 mg L^−1^ TC-HCl solution was prepared, with ultrasonic treatment for 20 minutes to ensure uniform dispersion of TC-HCl molecules. Photocatalyst Cu_3_P/SnO_2_ (50 mg) was uniformly dispersed in 40 mL TC-HCl solution. Then, stirring was continued for 30 minutes in the dark to make the TC and Cu_3_P/SnO_2_ sample reach the equilibrium of adsorption and desorption. After turning on the light source, 3 mL sample was taken every 20 minutes, and diluted with 3 mL distilled water. The diluted sample is centrifuged twice at 10 000 rpm to remove the sediment. The concentration of supernatant was determined by an UV-visible spectrophotometer at 360 nm, which is the typical absorption wavelength of TC-HCl. The photocatalytic process took for 140 minutes for several samples.

## 3. Result and discussion

### 3.1. Characterization analysis

**Fig. 1:**
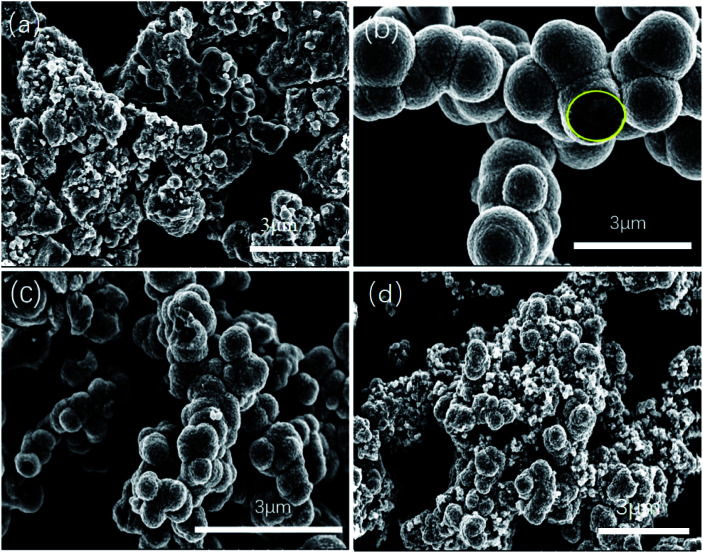
SEM images of the prepared catalysts of SnO_2_-1 : 3 (a), SnO_2_-1 : 4 (b) hollow sphere, SnO_2_-1 : 5 (c) hollow sphere, SnO_2_-1 : 6 (d).

SEM images (Fig. 1) were collected to observe the morphologies of SnO_2_. The SEM of SnO_2_ samples synthesized by hydrothermal method at different ration from 1 : 3 to 1 : 6 were displayed in Fig. 1a–d. The results showed that the SnO_2_-1 : 4 sample (Fig. 1b) exhibited the best morphology, consistent with the conclusions in previous work^44^ and the SnO_2_-1 : 4 sample possessed good photocatalytic activity. Additionally, average diameter is about 1 μm of the SnO_2_-1 : 4 sample, and each SnO_2_ 1-*X* microsphere exhibit a hollow structure with pore sizes ranging from 200 nm to 500 nm. The morphology of SnO_2_-1 : 5 samples (Fig. 1c) showed a hollow structure to some extent with different sizes. For the other two samples of SnO_2_-1 : 3 (Fig. 1a) and SnO_2_-1 : 6 (Fig. 1d), the hollow structure was obviously broken. The arrangement of SnO_2_ was irregular with small porosity, and the specific surface area and additional active sites increased and turned conducive to photocatalytic degradation.

**Fig. 2:**
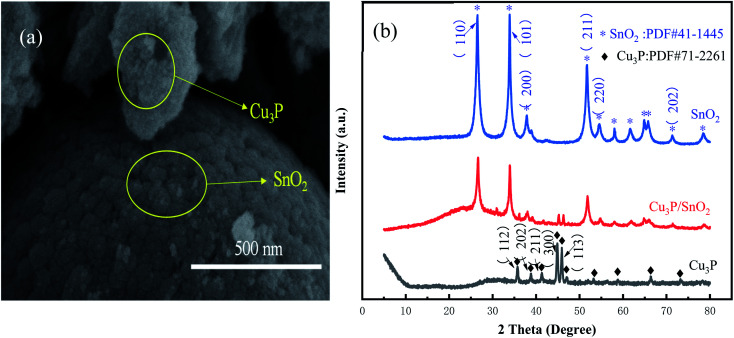
SEM images of Cu_3_P/SnO_2_ heterostructure (a) and XRD patterns of the prepared catalysts (b).

As shown in Fig. S1 in ESI,† Cu_3_P is very small in size, similar in shape to cotton mass with a diameter of about 250 nm. Fig. 2a demonstrated the morphology of Cu_3_P/SnO_2_ composites, SnO_2_ was a sphere with much larger size than that of Cu_3_P, with a diameter of about 1 μm. Cu_3_P nanoparticles were clustered on the surface of SnO_2_ microspheres, with a solid–solid interface formed between the two chemicals. These results demonstrated that Cu_3_P had been successfully installed on the surface of SnO_2_.

XRD patterns in Fig. 2b showed crystalline structures of the synthesized parent materials of Cu_3_P and SnO_2_, and the Cu_3_P/SnO_2_-3 heterostructure. From XRD pattern of SnO_2_, the weaker diffraction peaks located at 37.949°, 54.757° and 71.276° which belonged to the (200), (220) and (202) lattice planes of SnO_2_, while the stronger diffraction peaks located at 26.611°,33.893° and 51.780°, which is attributed to the (110), (101) and (211) lattice planes of SnO_2_ respectively. XRD pattern of SnO_2_ in Fig. 2 were keeping up with the standard card (JPDF no. 41-1445).

XRD pattern of Cu_3_P in Fig. 2 was consistent with the standard card (JPDF no. 71-2261), and the diffraction peaks were at 36.005°, 39.078°, 41.577°, 45.091° and 46.158°, which was corresponded to (112), (202), (211), (300) and (113) lattice planes of Cu_3_P, respectively. Besides, the purity of Cu_3_P was very high since there is no extra impurity peak. It is worth noting that both diffraction peak of Cu_3_P and diffraction peak of SnO_2_ were plotted in the XRD diffraction pattern (Fig. 2) of the Cu_3_P/SnO_2_ heterostructure, indicating the successful combination of Cu_3_P and SnO_2_.

Compared with the XRD peaks on the (110), (101) and (211) lattice planes (Fig. 2) of SnO_2_, the peak width of the Cu_3_P/SnO_2_ heterostructure became smaller, reflecting bigger average crystallite size. From Table 1, the average crystallite size of the (110), (101) and (211) lattice planes of SnO_2_ was 97, 130 and 121 nm. The average crystallite size of Cu_3_P and SnO_2_ in the composite increased to some extent. The average crystallite size of the (110), (101) and (211) lattice planes of Cu_3_P/SnO_2_ heterostructure was to 143, 168 and 138 nm.

**Table 1:** Average crystallite size of (110), (101) and (211) lattice planes of sample

Sample	Average crystallite size of (110) lattice plane (nm)	Average crystallite size of (101) lattice plane (nm)	Average crystallite size of (211) lattice plane (nm)
SnO_2_	97	130	121
Cu_3_P/SnO_2_	143	138	138

**Fig. 3:**
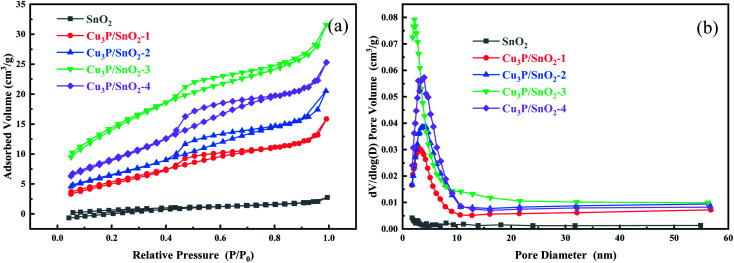
Nitrogen adsorption and desorption isotherms (a) and pore diameter distribution curves (b) of SnO_2_ and Cu_3_P/SnO_2_-1, 2, 3 and 4 composites.

The pore size and BET specific surface area were determined by N_2_ adsorption of the synthesized samples (Fig. 3). Fig. 3a showed the N_2_ adsorption and desorption isotherms of SnO_2_ and Cu_3_P/SnO_2_-1, 2, 3, and 4. According to the classification standard, the isotherm of the composite Cu_3_P/SnO_2_ at a relative pressure (*p*/*p*_0_) of 0.5–1.0 belonged to the IV type isotherm, and a typical H_3_ hysteresis loop. This verified that composite Cu_3_P/SnO_2_ heterostructure was a mesoporous material. Compared with pure SnO_2_, the BET specific surface area of the heterostructure Cu_3_P/SnO_2_-3 increased significantly. According to the BET theoretical calculation model, Table 2 was categorized with the specific surface area of pure SnO_2_ was 3.7892 m^2^ g^−1^, while the specific surface area of Cu_3_P/SnO_2_-3 was 53.1470 m^2^ g^−1^, which was about 14 times that of SnO_2_. Fig. 3b showed the pore size distribution curves of Cu_3_P/SnO_2_ samples. The pore size of Cu_3_P/SnO_2_ heterostructure was distributed around 5–20 nm. Based on BJH theoretical calculation model, the pore volume of SnO_2_ was 0.003904 cm^3^ g^−1^, and the pore volume of Cu_3_P/SnO_2_-3 heterostructure was 0.046550 cm^3^ g^−1^, with a tremendous increase of 12 times (Table 2). From Table 2, the specific surface area of Cu_3_P/SnO_2_-3 was the largest (53.1470 m^2^ g^−1^) while the average pore size was the smallest (3.8213 nm). The increase in the surface area were attributed to the surface modification of Cu_3_P nanoclusters on SnO_2_. The larger the specific surface area of the sample, the easier of the sample to expose active sites on the surface. As a photocatalyst, it increased the photocatalytic activity of the pollutant tetracycline (TC-HCl).

**Table 2:** Specific surface area and pore parameters of different samples

Sample	Specific surface area (m^2^ g^−1^)	Pore volume (cm^3^ g^−1^)	Average pore size (nm)
SnO_2_	3.7892	0.003904	5.4221
Cu_3_P/SnO_2_-1	20.2940	0.023992	4.7529
Cu_3_P/SnO_2_-2	24.3244	0.032098	4.5883
Cu_3_P/SnO_2_-3	**53.1470**	**0.046650**	3.8213
Cu_3_P/SnO_2_-4	33.9863	0.039631	4.2267

**Fig. 4:**
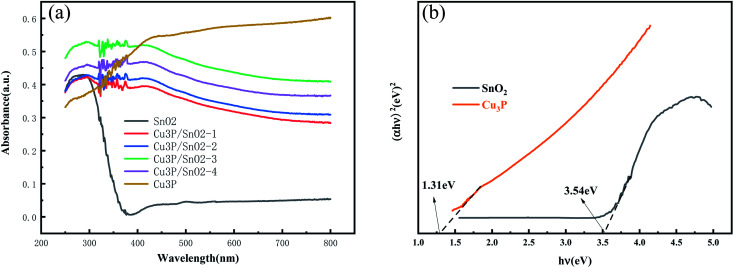
Ultraviolet-visible absorption spectra of SnO_2_, Cu_3_P and heterostructure Cu_3_P/SnO_2_-1, 2, 3 and 4 samples (a); the gap width of SnO_2_ and Cu_3_P samples (b).

In order to study the modification effect of mesoporous Cu_3_P nanoclusters on SnO_2_ microspheres to improve its photocatalytic performance, UV-vis DRS was applied to analyze absorption properties of SnO_2_, Cu_3_P and Cu_3_P/SnO_2_-1–4 heterostructure. Fig. 4a showed the UV-vis absorption spectrum of SnO_2_, Cu_3_P and heterostructure Cu_3_P/SnO_2_-1, 2, 3, and 4 samples. Cu_3_P has strong absorption strength in the whole wavelength range, especially in the visible light range. The SnO_2_ has a strong absorption intensity in range of 200–360 nm (UV), while there was negligible absorption intensity above 400 nm (visible). This indicated that SnO_2_ can only be excited by ultraviolet photons to undergo electronic transitions, without any optical response in the visible wavelength range. Compared with bare SnO_2_, the absorption intensity of Cu_3_P/SnO_2_ heterostructure was quite different, with a higher absorption intensity in the visible range of 400–800 nm, which was related to the modification effect of mesoporous Cu_3_P nanoclusters on the surface of SnO_2_ nanosheets. This indicated a strong interaction between Cu_3_P and SnO_2_, which was beneficial to improving the migration efficiency of electron–hole pairs and inhibited their rapid recombination. Furthermore, the band gap values were estimated by Tauc plots and plotted in Fig. 4b. From the Fig. 4b the *E*_g_ for Cu_3_P and SnO_2_ samples are about 1.31 eV and 3.54 eV, respectively.

**Fig. 5:**
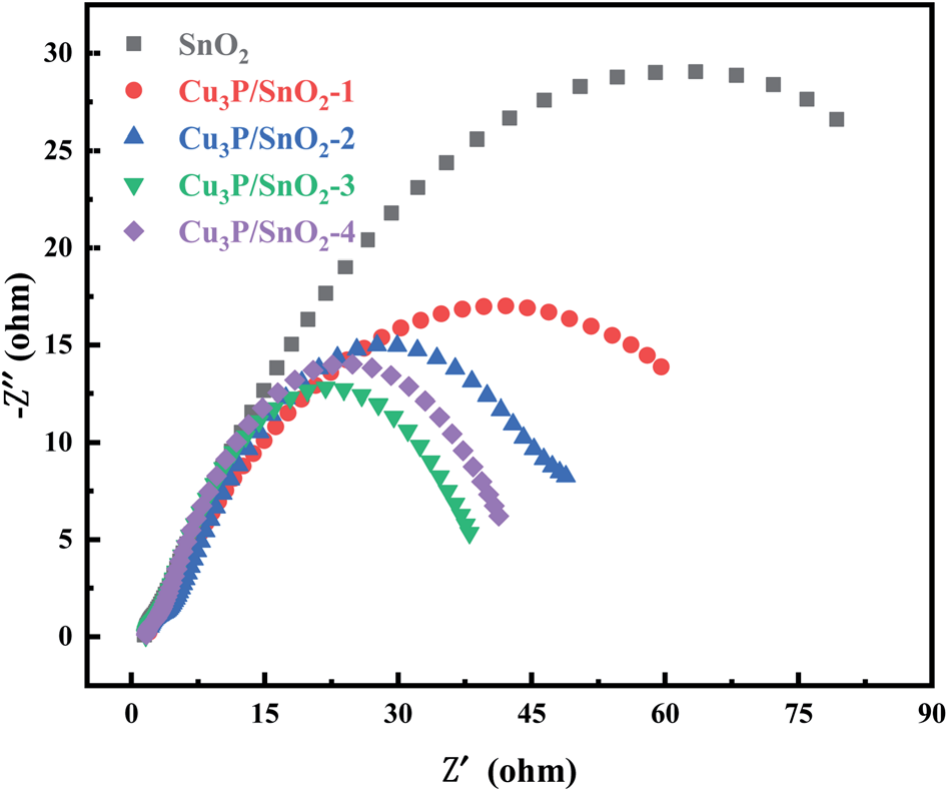
The Nyquist plots of SnO_2_ and heterostructure Cu_3_P/SnO_2_-1, 2, 3 and 4 samples.

Fig. 5 showed the EIS measurements of pure SnO_2_ and Cu_3_P/SnO_2_ samples. The arc size of the Nyquist plot of SnO_2_ were large due to its large energy gap. In the EIS Nyquist diagram, the smaller the arc size, the smaller the charge transfer resistance and the higher the efficiency of electron–hole separation. Based on previous publications of SnO_2_ materials,^44^ different studies reported different results, from 20 to 1250 ohms.^45,46^ From Fig. 5 the arc diameter of SnO_2_ was about 120, reflecting the charge transfer resistance Rct of 120 ohms. The Rct of Cu_3_P/SnO_2_-3 sample was the smallest (40 ohms), followed by the order of Cu_3_P/SnO_2_-1 (80 ohm) > Cu_3_P/SnO_2_-2 (60 ohm) and Cu_3_P/SnO_2_-4 (50 ohm), respectively. The results indicated that the optimal Cu_3_P loading is 30% wt. The tight solid–solid interface generated between the two semiconductors of Cu_3_P and SnO_2_ turned into a channel for charge transfer, which reduced the charge transfer resistance in interface and improved the migration rate of electric load current carrier.

### 3.2. Adsorption and photocatalytic activity evaluation

**Fig. 6:**
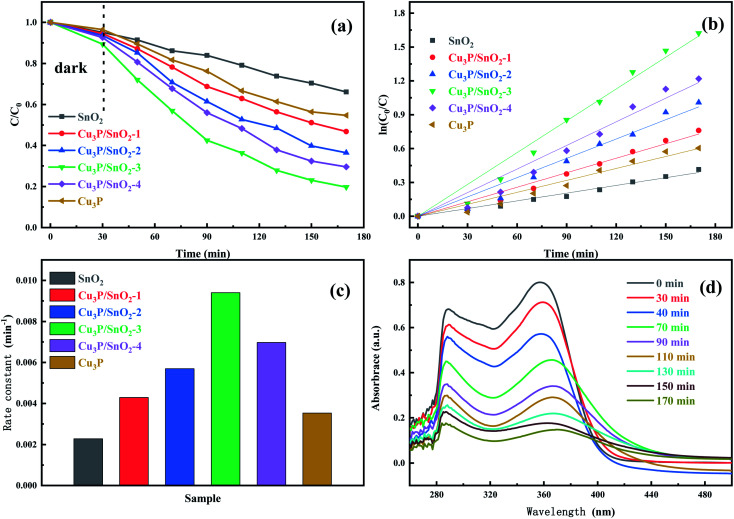
Photocatalytic degradation dynamic curves of SnO_2_, Cu_3_P and composite Cu_3_P/SnO_2_-1–4 under visible light radiation (a); first-order dynamics fitting curve(b); degradation rate constants of different samples (c); absorption spectra of tetracycline by Cu_3_P/SnO_2_-3 at different time points (d).

From the photocatalytic experiment, the photocatalytic activity was explored of different samples under visible light radiation. Fig. 6a showed the dynamic curves of SnO_2_, Cu_3_P and composite material heterostructure Cu_3_P/SnO_2_-1, 2, 3 and 4 in the photocatalytic degradation of tetracycline. The samples underwent dark reaction adsorption for 30 minutes, and then was irradiated under visible light for 140 minutes. The results showed that after adsorption equilibrium was achieved in the dark, the maximum adsorption capacity was 10% of the heterostructure Cu_3_P/SnO_2_-3, and the adsorption capacity of SnO_2_ sample was 5%, which indicated that the increase of physical adsorption capacity. Such difference was due to the morphological structure of Cu_3_P/SnO_2_ composite system increased their specific surface area.

In the 140 minutes visible light irradiation stage, the degradation effect on tetracycline was obvious. The degradation effects were different of heterostructure materials Cu_3_P/SnO_2_-1, 2, 3 and 4 with different composite ratios. The degradation efficiency SnO_2_ samples was only 35%, since SnO_2_ cannot fully absorb visible light, and ultraviolet light accounts for a small proportion of visible light. When Cu_3_P was introduced into the SnO_2_ material and Cu_3_P/SnO_2_ was generated, the photocatalytic degradation effect was significantly enhanced. The photocatalytic degradation effect of Cu_3_P/SnO_2_-3 became the best among rest samples and the degradation efficiency reaches 80%. From Fig. 6a, the order of degradation efficiency was: Cu_3_P/SnO_2_-3 (80%) > Cu_3_P/SnO_2_-4 (70%) > Cu_3_P/SnO_2_-2 (64%) > Cu_3_P/SnO_2_-1 (53%) > Cu_3_P(45%) > SnO_2_ (34%), the higher the better.

Fig. 6b showed the kinetic curves of Fig. 6a, which fitted to the first-order kinetic model ln(*C*_0_/*C*) = *kt*. The photo degradation process was first order reaction. Fig. 6c was a histogram of the rate constant *K* of degradation of tetracycline by SnO_2_, Cu_3_P and composite heterostructure Cu_3_P/SnO_2_-1, 2, 3 and 4. The rate constant *k* of pure SnO_2_ and Cu_3_P were 0.00228 and 0.00353 min^−1^, while the rate constant *k* of the sample Cu_3_P/SnO_2_-3 was the maximum (*k* = 0.00940 min^−1^), which was 4.1 times and 2.7 times than that of SnO_2_ (*k* = 0.00228 min^−1^) and Cu_3_P (*k* = 0.00353 min^−1^), indicating the Cu_3_P/SnO_2_ effectively enhanced the photocatalytic degradation efficiency. The rate constant *k* of the other samples Cu_3_P/SnO_2_-1, 2 and 4 were 0.00429, 0.00570 and 0.00697 min^−1^, respectively. These results verified that the optimal composite percent was 30% wt of Cu_3_P/SnO_2_ composite, with the best photocatalytic performance for tetracycline. In addition, Fig. 6d showed the absorption spectra of Cu_3_P/SnO_2_-3 for tetracycline at different times. With the gradual extension time of visible light radiation, the characteristic absorption wavelength of 360 nm of tetracycline absorption showed a significant downward trend. This indirectly confirmed that with increased period of visible light radiation, tetracycline gradually was decomposed and opens the ring to breakdown into small molecules or ions.

Table 3 categorized several photocatalysts that had been reported in literature. The results showed that Cu_3_P/SnO_2_-3 contained 80% degradation rates, which was comparable with different reported catalysts, such as FeOOH/FeS_2_ of 90%,^47^ Ag/Bi_3_TaO_7_ of 85% (ref. 48) and Bi_12_O_15_C_l6_/Bi_2_WO_6_ of 81%.^49^ This work is better than BiFeO_3_/TiO_2_ of 72% (ref. 50) and In_2_S_3_/InVO_4_ of 71%.^51^ Besides, the dosage (*W*_cat_/TC) of Cu_3_P/SnO_2_-3 was 25 mg mg^−1^, which was the minimum around the rest 5 types of catalysts around 50–100 mg mg^−1^. The amount of catalysts Cu_3_P/SnO_2_-3 used in photocatalytic experiment was much less with less pollution and lower cost. This indicates that the catalyst is more favorable to treat the wastewater containing relatively high tetracycline.

**Table 3:** Photo degradation effects of TC among different catalysts

Photocatalysts	Catalyst dosage (mg L^−1^)	TC concentration (mg L^−1^)	*W*_cat_/TC (mg mg^−1^)	Dark (min)	Illumination (min)	Removal rate	Reference
FeOOH/FeS_2_	500	10	50	30	150	90%	Guo *et al.*^47^
Ag/Bi_3_TaO_7_	1000	10	100	60	120	85%	Luo *et al.*^48^
Bi_12_O_15_C_l6_/Bi_2_WO_6_	500	10	50	30	60	81%	Wu *et al.*^49^
BiFeO_3_/TiO_2_	1000	10	100	60	180	72%	Liao *et al.*^50^
In_2_S_3_/InVO_4_	500	10	50	60	120	71%	Yuan *et al.*^51^
Cu_3_P/SnO_2_	1250	50	25	30	180	80%	This study

### 3.3. Stability and degradation mechanism studies 6tb

**Fig. 7:**
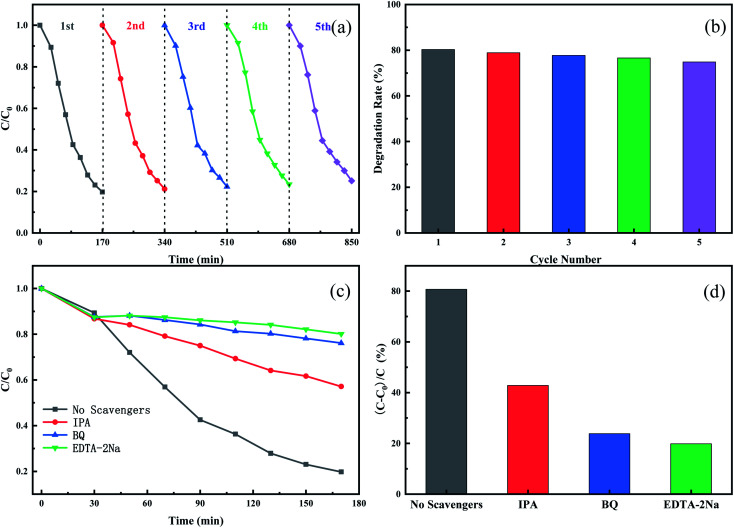
The cyclic degradation curve of tetracycline over heterogeneous Cu_3_P/SnO_2_-3 composite catalyst (a); column comparison of degradation rates (b); degradation dynamic curve after the addition of capturing agent (c); histogram of degradation efficiency of different capture agents (d).

To evaluate the stability and reusability of the Cu_3_P/SnO_2_ heterostructure as a photocatalyst, we conducted five cycles of photodegradation experiments on the Cu_3_P/SnO_2_-3 catalyst. After completing a cycle of photodegradation experiment, the catalyst was collected by simple centrifugation, and treated with rinse, drying and other operations. The collected catalyst was prepared for the next cycle experiment. Fig. 7a and b showed the degradation curves and removal efficiency of tetracycline after five cycles. After multiple photodegradation cycles, the degradation efficiency of tetracycline changed a little, from 80% to 75%. After 5 consecutive cycles, it only dropped by 5%. These results proved stability of Cu_3_P/SnO_2_-3 as a photocatalyst, and it can be recycled with treatment, which effectively reduced the cost of catalyst production.

The reaction mechanism of tetracycline breakdown was studied by adding active oxidant species capture agent in the photocatalytic reaction stage. Isopropyl alcohol (IPA), benzoquinone (BQ) and ethylene diamine tetra acetic acid disodium salt (EDTA-2Na) were used to capture hydroxyl radical (˙OH), photogenerated holes (h^+^) and superoxide radicals (˙O_2_^−^) of active oxidizing species, respectively. From the kinetic degradation curve (Fig. 7c) and the histogram (Fig. 7d) of degradation efficiency, the degradation of tetracycline by Cu_3_P/SnO_2_-3 catalysts was greatly different with different capture reagents. Results showed that the addition of active species capture agent did take into effect. After adding EDTA-2Na and BQ as capturing reagents for h^+^ and ˙O_2_^−^ to the photocatalytic reaction pathway, the degradation efficiency of TC-HCl is dropped down to 19% and 24%, much lower than the blank test of non-capturing reagents (80%). This strong inhibitory effect indicated that h^+^ and ˙O_2_^−^ played a key role in the degradation of tetracycline, and the effect of h^+^ was slightly higher than that of ˙O_2_^−^. However, if IPA was used as a capture agent for ˙OH, the degradation efficiency of tetracycline drops from 80% to 57%, indicating that ˙OH plays a minor role in the degradation of TC-HCl. In summary, in the process of tetracycline degradation by Cu_3_P/SnO_2_ composite, the role of h^+^, ˙O_2_^−^ and ˙OH follows h^+^ > ˙O_2_^−^ > ˙OH of the degradation reaction.

The ESR measurements were further used to identify ˙O_2_^−^ in Cu_3_P/SnO_2_-3 composite catalysts. As shown in Fig. S2 in ESI,† no signal was detected under dark conditions. Under visible light irradiation, ˙O_2_^−^ appeared obviously, which proved that the production of the active substance was consistent with the free radical trapping experiment. Therefore, Cu_3_P/SnO_2_ photocatalyst showed good performance with the help of reactive oxygen species.

**Fig. 8:**
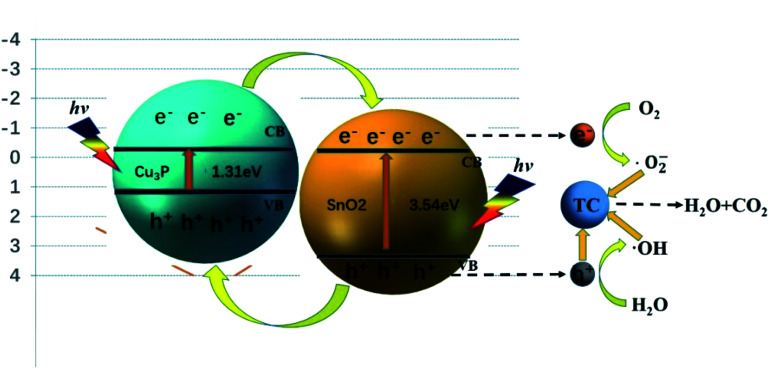
Mechanism diagram of photocatalytic degradation of TC by heterogeneous Cu_3_P/SnO_2_.

Fig. 8 showed the possible mechanism of the degradation of TC by heterostructure Cu_3_P/SnO_2_ catalyst under visible light. In the previous calculation, the band gap values of Cu_3_P and SnO_2_ were 1.31 eV and 3.54 eV, respectively. Under the visible light radiation, SnO_2_ and Cu_3_P are respectively excited, and the electrons in the valence band (VB) migrate to the conduction band (CB), leaving the hole h^+^ in the VB. Part of the electrons in the CB of Cu_3_P were transferred to the CB of SnO_2_ through the heterostructure interface. H_2_O molecules trap holes h^+^ in the VB and convert them into ˙OH, and part of the oxygen O_2_ molecules adsorbed on the catalyst surface trap electrons e^−^ in the CB and converted them into superoxide radical ˙O_2_^−^. Finally, the holes h^+^, ˙O_2_^−^ and ˙OH directly oxidize the tetracycline TC molecules. This was the general degradation principles of tetracycline TC by the studied Cu_3_P/SnO_2_ heterostructure. The specific and detailed degradation mechanism of tetracycline molecules await future research.

## 4. Conclusion

Cu_3_P/SnO_2_, the p–n type heterostructure, was proved to be an efficient photocatalyst for the degradation of antibiotic drug pollutants like TC-HCl. Among different Cu_3_P loaded samples, Cu_3_P/SnO_2_-3 (30% load) after 30 minutes of dark reaction adsorption and 140 minutes of photodegradation, the removal efficiency reached 80%, which is 2.3 times of the removal efficiency of SnO_2_. The improved photocatalytic activity was benefited from the heterogeneous interface between SnO_2_ and Cu_3_P, which effectively enhances the efficient charge transfer and retarders the recombination of electron hole pairs. Furthermore, the cyclic photodegradation experiments verified that the heterojunction Cu_3_P/SnO_2_-3 was stable and the efficiency dropped 5% after 5 runs. From mechanistic study, the active oxidant species as h^+^, ˙O_2_^−^, and ˙OH were all involved in photocatalytic system. It seems that h^+^ and ˙O_2_^−^ played the major role in the photocatalytic degradation of tetracycline by Cu_3_P/SnO_2_ composites. Further work may involve the improvement of catalyst with better degradation activity.

## Conflicts of interest

There are no conflicts to declare.

## Supplementary Material

RA-011-D1RA05905J-s001
