# Supplementary material for: Synthesis of Cu3P/SnO2 composites for degradation of tetracycline hydrochloride in wastewater
Source: RSC Adv. 2021 Nov 12;11(53):33471–80. doi: 10.1039/d1ra05905j (PMC9042268; doi:10.1039/d1ra05905j)
Supplement: RA-011-D1RA05905J-s001 [file RA-011-D1RA05905J-s001.pdf]

Electronic Supplementary Material (ESI) for RSC Advances.
This journal is © The Royal Society of Chemistry 2021

Support Information：

SEM image of Cu3P and ESR/EPR figures of radicals.

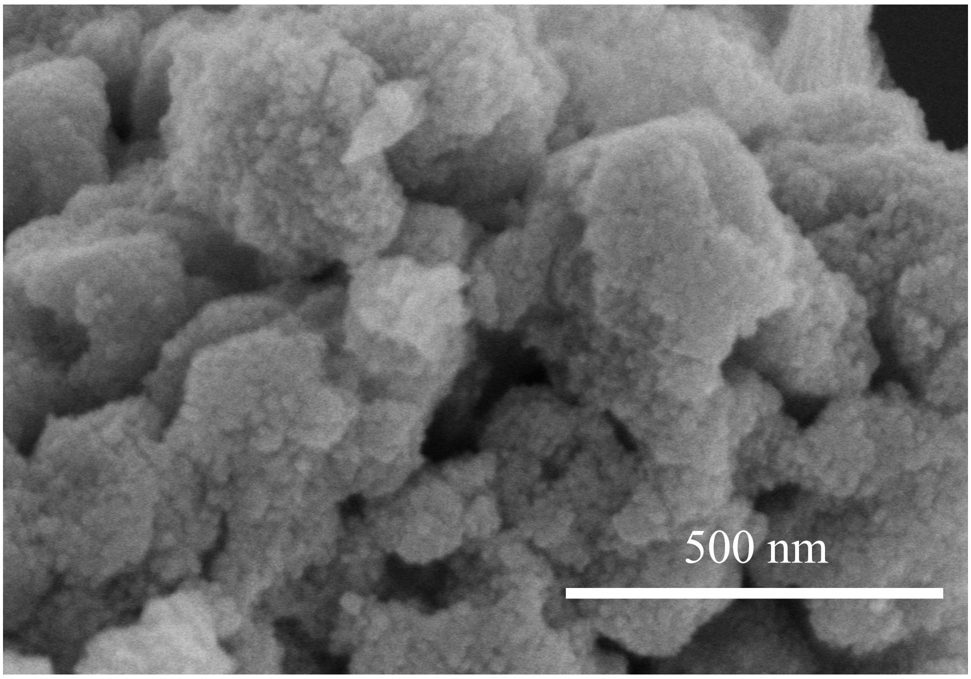

Fig S1. SEM images of the prepared catalysts of Cu_3P

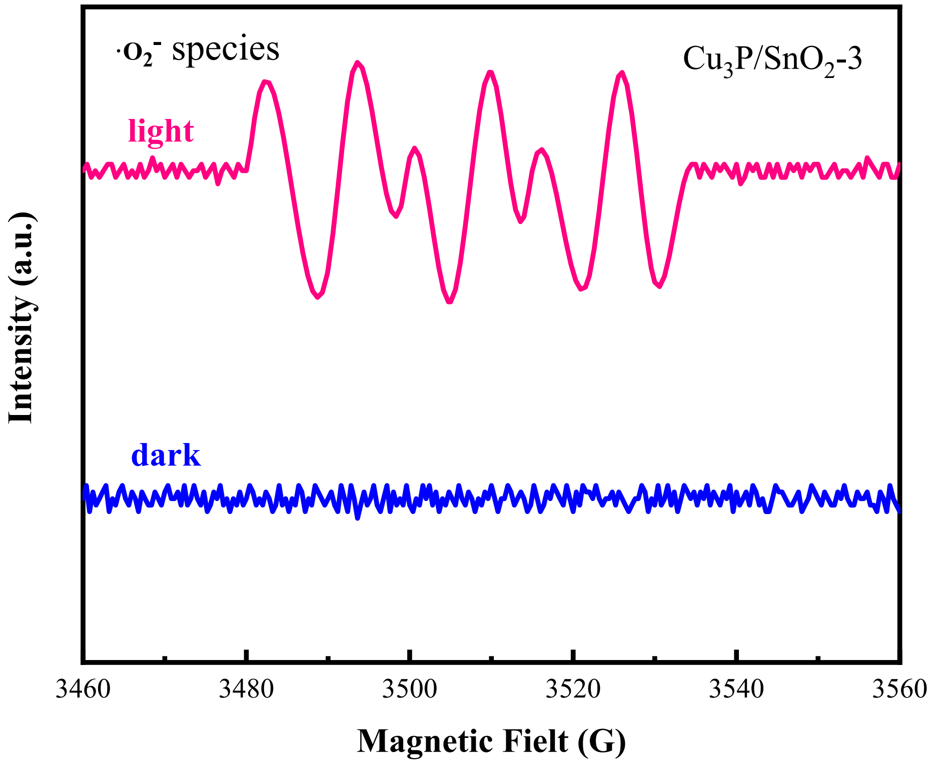

Fig. S2 ESR tests for the $\cdot O_2^-$ species during different catalytic processes
